# Gender and intersectional analysis of livestock vaccine value chains in Kaffrine, Senegal

**DOI:** 10.1371/journal.pone.0252045

**Published:** 2021-07-01

**Authors:** Sarah McKune, Renata Serra, Alioune Touré

**Affiliations:** 1 Department of Environmental and Global Health and Center for African Studies, University of Florida, Gainesville, FL, United States of America; 2 Center for African Studies, University of Florida, Gainesville, FL, United States of America; 3 Department of Livestock Science & Technology, Université du Sine Saloum El Hadj Ibrahima Niass, Kaffrine, Senegal; University of Minnesota, UNITED STATES

## Abstract

Among livestock species, poultry and small ruminants are of particular importance to rural women in low- and middle-income countries, as means to generate income, provide nutritious food for the family, accumulate wealth, and confer social status. Newcastle disease (ND) and Peste des Petits Ruminants (PPR) are widespread livestock diseases of poultry and small ruminants, respectively. While both diseases are vaccine preventable, numerous constraints limit the availability of and access to livestock vaccines, especially among the most vulnerable populations in developing countries. The literature on equity and effectiveness of livestock vaccine distribution systems has emphasized many of these constraints, however a gendered analysis and deeper understanding of the vaccine system remain insufficient. This paper applies a *gendered and intersectional transformational approach*, or GITA, to highlight how gender and other social factors affect the provision and utilization of vaccines for ND and PPR diseases in the region of Kaffrine, Senegal. We first articulate and describe the vaccine value chains (VVCs) for these diseases in Kaffrine, and then analyze the gendered and intersectional dynamics at different nodes of the VVCs, including actors at the national level, through the regional and district levels, down to providers of animal health at community level and the livestock keepers themselves. Our findings indicate that actors’ various experiences are shaped and defined mainly by rigid gender norms, location and remoteness, and to a lesser degree by other social stratifications of age, ethnicity, and livelihood. Given the significant role that gender norms play in the livestock vaccine value chains, differences according to the livestock species, regulation of vaccine administration, and vaccine distribution systems emerge as highly relevant for understanding barriers that women specifically face within the livestock vaccination system.

## Introduction

Livestock play an essential role in the overall wellbeing of smallholder households across low- and middle-income countries. Animal health is inextricably linked to human health, both through zoonotic diseases, as well as the negative social and economic consequences of animal diseases on people whose livelihoods are reliant on livestock. Among livestock species, poultry and small ruminants are of particular importance to rural women as a means to generate income, provide nutritious food for the family, accumulate wealth, and confer social status. Newcastle disease (ND) and Peste des Petits Ruminants (PPR) are important livestock diseases of poultry and small ruminants, respectively, and are among the most diffused and deadly livestock diseases experienced by smallholder farmers, particularly women [1]. They are also vaccine preventable diseases. The provision of livestock vaccines is essential to help curb livestock diseases, prevent the transmission of zoonotic infections to humans, enhance animal sourced food quality, and ultimately contribute to sustainable livelihoods [2]. While most governments in low- and middle-income countries have invested heavily in vaccination interventions, the realities on the ground are complex and varied, with numerous constraints limiting the availability of and access to livestock vaccines, especially among the most vulnerable populations [3, 4].

The literature on the equity and effectiveness of vaccine distribution systems has emphasized many of these constraints, however a gendered analysis and deeper understanding of the vaccine system remain insufficient [5, 6]. Challenges to livestock vaccination systems are due to both supply and demand side factors and involve technical, logistical, and financial issues, as well as socio-economic and psychological barriers [7]. Livestock keepers in poorer countries are found to have limited demand for vaccines due to cost and distance, but also lack of trust in the system and safety worries [8]. The willingness to pay for vaccines and the willingness to vaccinate also depend on the degree of information about the vaccination campaigns and the market orientation of livestock keepers [9]. While some of these studies recognize that gendered factors play an important role in determining individuals’ ability to operate in the vaccine distribution system and/or access livestock vaccines, few of them disaggregate data by sex and, if they do, the scope of gender analysis remains limited. It would be instead important to examine how the sex of the livestock owner and gender relations and norms at the household and community level affect incentives, barriers and outcomes for livestock owners and other actors in the livestock vaccine distribution system.

In addition to gender, there are numerous social stratifications–those societal dynamics which serve to group, divide, distinguish, or otherwise characterize groups of people–that affect an individual’s ability to engage in the livestock distribution system, though this evidence base is scant. Theories of intersectionality acknowledge that forms of inequality are mutually constitutive and that sources of oppression are overlapping and dynamic [10–13]. In public health, intersectionality has been used to unpack how race, gender, ethnicity, and other social factors interact to marginalize and disadvantage certain individuals not additively, but synergistically [14–16]. Despite significant growth in the field of One Health, a public health approach built on the recognition that the health of people is intrinsically connected to the health of animals and a shared environment [17], the role of intersectionality in One Health is not often fully investigated.

This paper addresses these gaps by applying a *gendered and intersectional transformational approach*, or GITA [18], to the study of vaccine distribution systems in order to identify both the distinct gender roles and gendered constraints arising at different points within these systems, as well as compare how such gender norms and roles may affect the provision and/or utilization of vaccines differentially for poultry and small ruminant diseases, taking the case of the region of Kaffrine, Senegal. First, we conceptualize the vaccine space through the analytical tool of vaccine value chain (VVC), an extension of the more general concept of agricultural value chains [19, 20]. As in agricultural value chains, VVCs are complex systems entailing actors and activities at different and connected nodes. Along the VVC, vaccines move between different geographical, institutional, and social spaces, typically from the national to the regional and finally to the local community level. At each node designated actors are expected to safely store the vaccine and/or administer it to livestock throughout the territory.

Our second contribution is to apply the GITA lens at each subsequent node of the VVC, in order to identify and examine the different constraints and opportunities that actors face engaging in or with the VVC, according to their gender, age, geographic location, livelihood and social status. We build on both research that analyzes agricultural value chains through a gender lens [21, 22] and literature that applies gender transformative approaches to development and health interventions more widely [23–26]. While gender integration into livestock vaccine supply chain is garnering attention [5], literature that engages intersectionality in examining factors affecting vaccine distribution or use distinguishing by value chain node and livestock species is rare and much needed [27, 28]. By relying on our comparative approach, we find that indeed gender norms and roles affect the provision and utilization of vaccines in different ways. In particular, differences due to the livestock species, regulation of vaccine administration, and vaccine distribution systems are more relevant for understanding barriers within livestock vaccination systems faced by women rather than by men.

The paper is organized as follows. After introducing the research methods and approach, results are presented in two parts: articulation and presentation of the vaccine value chains, and analysis of their gendered and intersectional dynamics at different nodes of the value chains. A discussion section points to how the identified barriers differ by value chain and vaccine type, highlighting the paper’s key contributions to the literature and gendered analyses of VVCs. We end with brief conclusions.

## Methods

### Study area

Senegal is located on the westernmost coast of West Africa. While a lower middle-income country and with a relatively diversified economy by regional standard, over half the population is rural and depends on agricultural activities, including livestock, for a great portion of their livelihoods.

The research was conducted in the Kaffrine Region of Senegal. Located southeast of Dakar, Kaffrine is one of 14 regional administrative units in Senegal, comprised of four departments: Birkilane, Kaffrine, Koungheul, and Malem Hodar. Known historically as the peanut basin of Senegal, many of the mixed livestock-crop producing households in the region produce either peanuts or fruits and vegetables as cash crops, in addition to growing millet and taking care of cattle, small ruminants, and poultry. The northern and eastern stretches of the region include the dry season home for pastoral populations who move large herds of cattle and small ruminants north to Linguere and the Ferlo region during the rainy season.

### Data collection

Methods used to document the VVC and to analyze the gender and intersectional dimensions of the VVC in Kaffrine included document review, key informant interviews (KIIs), individual interviews (IIs), and focus group discussions (FGDs). Each method is described in more detail below. Fieldwork data were collected between July and October 2019 under the guidance of an in-country coordinator. The researchers collected data in two phases, first in July/August, through a team of two male US-based graduate students (one Haitian and one Senegalese) with assistance from two Senegalese undergraduate students (one male, one female); followed by a second phase in September/October 2019, with a team of three male Senegalese field enumerators. The recruited students had either animal sciences or social science backgrounds.

All data were collected in the participants’ local language. All data collectors spoke French, and all but one spoke Wolof, the main language in the region. Instruments were developed in English, translated into French, reviewed and approved by University of Florida’s Institutional Review Board (IRB), protocol number 201901128 and administered orally with participants. Verbal informed consent was given by all participants, per accordance with the researchers’ university approved IRB protocol. The breakdown of KIIs, IIs, and FGDs by department and by gender is indicated in Table 1.

**Table 1:** Fieldwork data by collection method, location and gender, Kaffrine 2019.

Region	Department (Communes)	Key Informant Interview			Individual Interview			Focus Groups		
**Dakar**	**Dakar**	**Tot**	M	F	**Tot**	M	F	**Tot**	M	F
		5	4	1	0	0	0	0	0	0
**Kaffrine**	**Birkelane**	1	1	0	5	1	4	7*	4	2
	**Kaffrine**	12	8	4	20	12	8	15	8	7
	**Malem Hodar**	2	1	1	2	1	1	5	2	3
	**Koungheul**	3	3	0	3	1	2	4	2	2
**Total **		**23**	17	6	**30**	15	15	31*	16	14

Source: Senegal team’s fieldwork notes and transcripts, Summer/Fall 2019.

*1 additional mixed male/female FGD.

#### Document review

Throughout the preparation and implementation of the fieldwork, the UF-based team conducted a document review of peer-reviewed literature, institutional reports, and project reports. Relevant documents were identified through internet searches as well as by reference from experts and informants during the fieldwork.

#### Key informant interviews

Fieldwork was initiated with semi-structured interviews of purposively selected experts from the public and private sector, government, and international donors. Key informants were interviewed in one-on-one meetings by a member of the field team to identify the main actors, entry points, and distribution sites for livestock vaccines, as well as to improve understanding of the vaccine value chain and vaccination practices. Some of these interviews occurred in Dakar, where actors at national level provided an overall view of the vaccine distribution systems in Senegal. The in-country coordinator was critical to making email and phone introductions and connections to the initial key informants; a snowball sampling technique then included other actors in the VVC who were subsequently interviewed. Overall, the team conducted a total of 23 KIIs.

#### Individual interviews

Individual interviews (n = 30) were conducted with actors at the community level throughout the region of Kaffrine, including animal health workers and livestock keepers. These interviews aimed to capture their roles at the community level and their decision-making power related to the use of vaccines. Socio-demographic data on participants was collected to capture barriers and intersectionality at the lower nodes in the vaccine value chains.

#### Focus group discussions

To engage further the perspectives and experiences at the community level, focus group discussions (FGD; n = 31) were conducted with separate groups of men and women. Facilitators used open-ended questions about livestock keeping, livestock diseases, access to vaccines, and acceptance of vaccines to understand various aspects of VVCs. One member of the research team facilitated the discussion, while at least one additional member took notes to document this discussion. The information collected throughout these discussions contributed to a better understanding about the different roles and responsibilities of men and women in the respective VVC, as well as the identification of the elements of intersectionality (gender, ethnicity, livelihood, socio-economic class, etc.) that most affected participation in the VVC. During notetaking, attention was given to the interactions between the participants in relation to livestock vaccination practices at the community level.

### Data analysis

Findings presented here come from analysis conducted in three consecutive steps. Prior to fieldwork, an initial phase consisted of documenting the ND and PPR VVCs in Senegal, based on their characterization in the literature. This preliminary description served as a point of departure for the team, which informed the development of the KII, II, and FGD instruments as well as the identification of the target audience for data collection.

The second phase consisted in refining the description of the VVCs, based on the primary data collected in the field, which enabled us a more detailed understanding of the VVCs, the actors and activities at each node. This phase is documented and reflected in the final maps of the ND and PPR VVCs, as presented below.

**Fig 1:**
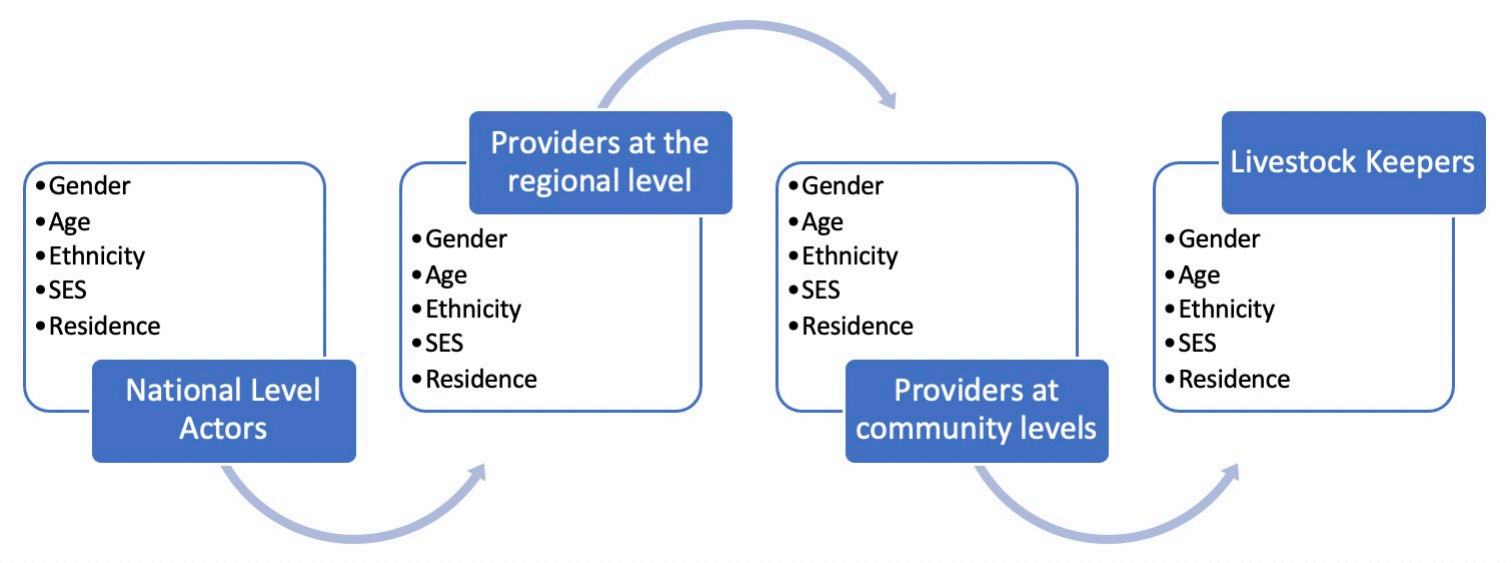
GITA approach to livestock vaccine value chain.

Finally, using the final VVC maps, the research team analyzed data from all primary data collection methods (KII, II, and FGD) to understand how gender, geographical location, education, livelihood, and age intersect to constrain or facilitate individuals’ engagement in and benefit from the VVC. At this phase, we employed a framework for analysis, established at the outset of this research, which we call GITA (gender intersectional transformative approach). GITA borrows from the latest gender analytic approaches in the health and development literature. By incorporating the notion of intersectionality in our understanding and investigation of gender, each individual or group is characterized by a set of multiple and overlapping social markers that activate and become relevant according to the circumstances. GITA is also informed by the notion that societal changes can only occur through a transformative approach that questions and challenges existing norms and beliefs [29]. Fig 1 illustrates merely how our GITA approach applies to a generic vaccine value chain: the data available at each node are investigated to elicit information on how gender and other intersections (including their socio-economic status, SES) together affect the distinct experiences of actors at a particular node of the value chain as well as the specific barriers they face.

The research team utilized this approach to analyze the KIIs, IIs, and FGDs. To start, researchers reviewed all transcripts and produced a summary report, identifying themes among each node was developed collectively. On that basis, a matrix was designed using Microsoft Excel, containing columns representing general information about the data (researcher, research methodology, actor, node), intersectional elements (sex, location, education, and livelihood), as well as men’s roles, women’s roles, constraints, and quotes. The research team again reviewed transcripts and pulled in relative information from each transcript (each transcript represented by one row in the Excel matrix). Findings were then integrated into the final map of the VCCs and are synthesized by node.

In the Results section below, the final map of the VCCs is presented with important differences between VVCs highlighted, followed by node-specific findings from the GITA analysis. Though KIIs were more informative of dynamics at higher nodes of the VVCs and FGDs were primarily informative of dynamics at lower nodes, the data generated by KII, II, and FGD all collectively contribute to a richer and more comprehensive thematic analysis of social dynamics and actors’ constraints across the nodes.

## Results

This section presents first a detailed analytical and visual description (mapping), which to our knowledge is the first of its kind in the published literature, of the vaccine value chains for PPR and ND in Kaffrine. We then apply the GITA tool along the VVC, starting at the higher nodes (national, regional and departmental levels), following with the key providers of animal health and vaccines at the community level and then concluding with the livestock keepers themselves.

### The mapping of PPR and ND VVCs in Senegal

Livestock VVCs in Senegal share a similar institutional approach and structure, which reflect the country’s legal and regulatory provisions. As the government of Senegal complies with international regulations, conventions, and agreements, at the top of the vaccine value chain several international actors play an important role in the country’s vaccine system, including the World Organization for Animal Health (French acronym, OIE), The Pan African Veterinary Vaccine Centre of African Union (PANVAC), and Food and Agriculture Organization of the United Nations (FAO). The government works with these international partners to monitor livestock diseases, ensure quality control of the vaccines, and train animal health professionals.

The Ministry of Livestock and Animal Production (French acronym, MEPA) oversees the regulatory framework for, and the coordination of, all interventions related to animal health and the prevention of livestock diseases throughout the national territory. This includes the approval and regulation of vaccines and the supervision of all actors in the distribution system. MEPA has prioritized five livestock diseases for eradication, which are PPR, ND, Contagious bovine pleuropneumonia, Lumpy skin disease, and African horse sickness. For each of these diseases, MEPA assures the regular administration of the vaccine during an annual campaign throughout the national territory.

Our analysis focuses on two priority diseases (PPR and ND) and the three vaccines against these diseases that are primarily used by smallholder livestock keepers, which are: the PPR/H live vaccine against PPR, administered through injection; the I2 vaccine against ND, a thermostable vaccine administered via drops in the eyes; and the ITA-new vaccine against ND, an attenuated live vaccine. As a result, we analyze three VVCs.

**Fig 2:**
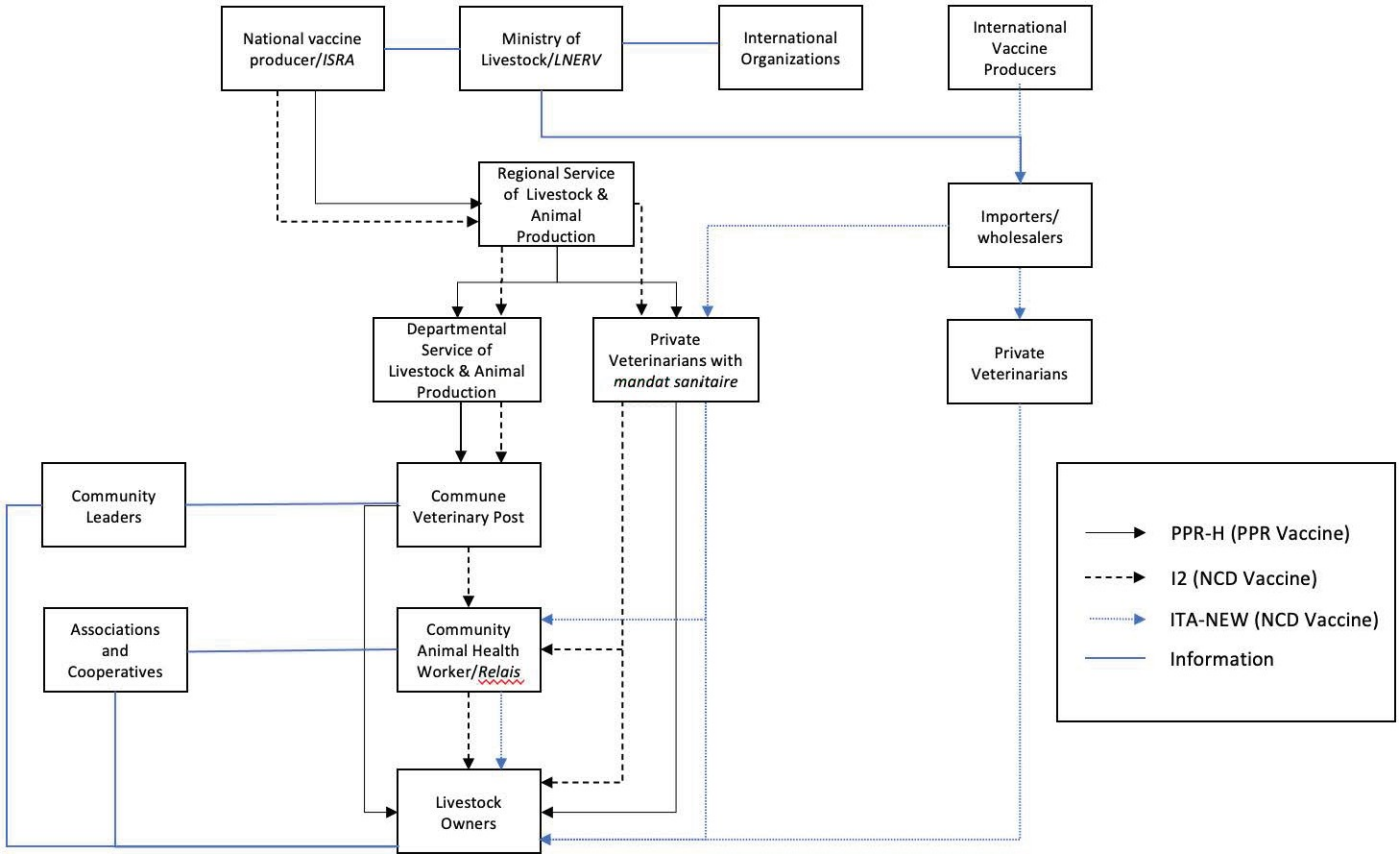
Mapping of PPR and ND vaccine value chains, Senegal.

One main difference between VVCs is whether they follow the public or the private distribution system, which are the two main livestock vaccine distribution systems in Senegal. The public distribution system applies to vaccines that are domestically produced and are funded and distributed by the state during an annual vaccination campaign. This system characterizes the VVC for both PPR/H and I2. The private distribution system is instead for all vaccines that are imported from foreign labs and distributed by private actors. The details for each of the three VVCs are described below and graphically represented in Fig 2.

#### Public distribution system: Value chains for PPR/H and I2

There are many similarities between the VVCs for PPR/H and I2. Both vaccines are produced by the National Livestock and Veterinary Research Laboratory (*Laboratoire National d’Elevage et de Recherches Vétérinaires*, *LNERV*), which is part of the Senegalese Institute for Agricultural Research (Institut Sénégalais de Recherches Agricoles, ISRA), the national agricultural research organization (see National vaccine producer/ISRA in Fig 2). LNERV/ISRA is the only vaccine producer in the country. Before the start of the vaccination campaign, MEPA places an order with LNERV/ISRA for the estimated required number of vaccine doses. Vaccines are then transported down through the public veterinary system, which is embedded within a broader livestock and animal production system, as follows:

- The Regional Service of Livestock & Animal Production is charged, among other functions, with enforcing and overseeing all veterinary functions on behalf of the state. For the purposes of this analysis, the collection of veterinary functions out of this office will be referred to as Regional Veterinary Services.
- The Departmental Service of Livestock & Animal Production, serve the four departments located in the region of Kaffrine, those being the departments of Kaffrine, Birkelane, Malem Hodar, and Koungheul. One of their key functions is to assess the number of animals by species in their territory and provide an estimate of the number of vaccines needed. This information is relayed to the Regional services, so that an order can be placed with the Directorate of Veterinary Services within MEPA ahead of the vaccination campaign. Similar to the regional level, for the purposes of this analysis, the collection of veterinary functions out of this office will be referred to as Departmental Veterinary Services.
- Veterinary Posts represent the public veterinary system at the level of communes (municipalities correspondents to small towns or regrouping several rural villages). The Heads of these posts are trained as ’livestock technical agents’, having graduated from the National Training Center for Livestock and Animal Industry Technicians (Centre National de Formation des Techniciens de l’Elevage et des Industries Animales, CNFTEIA), situated in Saint-Louis. Among other functions, they execute the vaccination campaigns and provide recommendations to livestock keepers. The number of Veterinary Posts in each department varies. According to our informants, there are four Heads of posts each in the departments of Birkelane and Malem Hodar, 6 in Kaffrine, and 8 in Koungheul, for a total of 22 in the region. At the moment of writing, there is one additional post unfilled in Kaffrine.
- In addition to c) above, in some areas, MEPA has issued a health authorization or permit (in French, *mandat sanitaire*) to private veterinarians. As established by a 1995 law, this permit gives them legal priority to administer vaccines on behalf of the state during the official vaccination campaign. These veterinarians with the government authorization/permit are paid, for each dose of vaccine administered, a small vaccination fee from the livestock keepers and are additionally compensated by the state. The vaccine is still provided by the government.

At the bottom of the VVC there is a diverse set of community animal health workers or agents, referred to locally with various French terms such as *auxiliaires* and *relais*–henceforth referred to solely as relais. These individuals have often received short training through a veterinary expert, an NGO, or a development project, and are equipped to perform several animal health-related services, including the administration of some vaccines. Relais are not public employees but are self-employed individuals, who get paid for the services they provide directly by the livestock keepers and, sometimes, by private veterinarians, if performing a service on their behalf.

One main difference to note stems from a Senegal law, which precludes individuals without at least the qualification of livestock technical agent from administering live vaccines, such as PPR/H. As a result, community level animal workers (the relais) are allowed to administer vaccines to poultry animals but not to small ruminants, creating in practice a gap between the VVCs for PPR (PPR/H) and ND (I2 or ITA-new).

#### Private distribution system: Value chain for ITA-new

The ITA-new vaccine against ND is produced by a foreign laboratory (LAPROVET) and imported by large private companies in Senegal, which specialize in the import of livestock products and act as wholesalers. While in the hands of private actors, vaccine imports are still subject to regulation by the state and require MEPA’s authorization. Imported vaccines are distributed through the private system, with importers/wholesalers selling to private veterinarians, each of whom has their pharmacies and sale points, serving the public directly or through the same relais that work in the public distribution system with technical livestock agents (see Fig 2).

### GITA analyses

While the livestock vaccine distribution system is well designed, it does not always work as it is supposed to. Interviews with actors in the VVC at various nodes, from the national to the local level, emphasized the existence of many constraints. The aim of this section is to show the gendered nature of such constraints, when applicable, and how these operate differently according to where the actors are situated in the VVC and to other intersectional aspects of their identity. Hence the GITA analysis that follows is organized by groups of nodes in the VVC, distinguishing between higher, middle and lower levels.

#### Higher-level nodes of the VVCs in Kaffrine

The highest node of the VVCs is at the central level in Dakar, where decisions about which vaccine to produce or to import, and in which quantities, are made every yearInformation about constraints to (women’s) engagement in the VVC at this level was derived largely from interviews with key informants (ISRA, the Ministry of Livestock, importers, and private sector companies). Possibly due to the limited presence of women at higher level (four of our five key informants were men), gender factors were not explicitly mentioned. Instead, it appears that relevant issues that preoccupy actors at this level were of technical and/or logistical nature. The main constraint emphasized by livestock personnel at the upper nodes of the VVCs is the disruption in vaccine procurement, which, in the case of the I2 vaccine, is related to limited access to Specific Pathogen Free eggs, which are an essential inputAnother constraint to increased production (applicable in the case of PPR/H) is posed by the requirement of freeze dryers. Bottlenecks and interruptions in the production of the vaccine were blamed for delays in vaccine orders, transport, and distribution further down the system. Key informants pointed out that any interruption in the production and/or administration of vaccines is doubly deleterious, since it potentially reduce vaccine effectiveness and thus fosters skepticism about the benefits of vaccination among livestock keepers.

#### Middle nodes of the VVCs

The middle nodes of the VVCs include the regional and departmental levels. These are key nodes where the vaccines are supposed to be stored safely and made promptly available during the vaccination campaigns. The interviews with veterinarians in the public and private distributions systems and the livestock technical agents pointed to two sets of constraints at this level–pertaining respectively to logistical bottlenecks and lack of women’s representation.

*Too wide catchment areas and lack of cold chain*. The geographical dispersion of the population, the size of the catchment areas and the limited number of posts equipped with suitable refrigeration systems are identified as key challenges to the effective and inclusive distribution of vaccines. Veterinary posts have only coolers to preserve the cold chain and there are only 22 of them in the whole Kaffrine region. The Heads of two Veterinary Posts, in Gnibi and Boulel, for instance, are supposed to cover, respectively, 21 and 42 villages. Not surprisingly, the heads of Veterinary Posts we interviewed repeatedly complained about the excessive demands of their jobs, and the lack of the necessary resources–motorbikes and relais–to serve the population efficiently and effectively.

*Overall lack of female representation*. Apart from the Head of Regional Veterinary Services being a woman at the moment of writing, there are not many other women veterinarians or technical agents involved in the veterinary services. Out of 22 technical livestock agents in the Kaffrine region, only four are women. This is attributed to both the limited upward mobility Senegalese women face, as well as the restrictive gender norms. The power of social norms is demonstrated in the explanation given by one of the female relais informants. In describing why women do not serve in higher-level positions, she said, “Women are generally found at the consumer level [of vaccines] … It will be difficult for beneficiary women to move to other nodes along the livestock vaccine value chain, because this would require intellectual capacities that they do not have” (Interview, female relais).

#### Lower nodes of the VVCs in Kaffrine: Animal health providers

At the community level, we collected data from both animal health providers (relais) and from livestock keepers themselves. Both play important roles and reflect different intersectional experiences, so we have divided findings from the lower nodes into these two groups. Community relais have an important role in that they connect the livestock keepers to veterinarians or Heads of Veterinary Posts. The relais may be men or women, and though some serve as community relais for particular livestock diseases, such as a PPR or ND relais, many serve more generically as community relais, thus are utilized by both the public and private vaccine distribution systems, as well as by livestock development projects. Themes that emerged from GITA analysis of this relais are presented below.

*Women have less access to training and education*. Some projects include women in training programs, some offering opportunities for them to become relais, others simply offering gender in agriculture training. By and large, trainings are highly valued by community members, men and women. However, women’s limited education constrains the degree to which they succeed in leveraging such opportunities. Adult literacy rates in Senegal are 40% for women and 65% for men (according to UNESCO data from 2017). Limited literacy and numeracy reduce women’s ability to systematically document their activities, productivity, and impact–endeavors that are advantageous if not essential for relais. Since trainings are found to be transformative by participants of either sex, the failure to successfully recruit women as vaccinators or relais has the potential to further marginalize women in this system. In one focus group, participants indicated that “no matter who you are, if you go through a training, you will come out a different person”. Some female relais report that the opportunity of attending seminars and trainings has changed the way they communicate with their husbands, improving their ability to garner support from them. Unfortunately, since projects reportedly target areas that are closer to the road and to fuel sources, communities that are geographically isolated hardly benefit from these opportunities and remain further marginalized. Women in isolated rural communities tend to be even more penalized in terms of training opportunities, given they face additional, gender-specific mobility constraints when compared to men. In addition to training, a general lack of knowledge about the importance of livestock vaccination was also cited, especially by animal health service providers, as an important barrier that limits some livestock keepers from benefitting from improved animal health. To the extent that rural women in Kaffrine tend to have lower access to formal and informal education, as well as other informal sources information, this problem is likely to affect women more than men.

*Gender norms limit women’s success as a relais*. Gender norms can significantly limit women’s ability to succeed as a relais. For example, access and use of a motorbike, which greatly facilitates the effectiveness and efficiency of a relais in doing his/her job, is culturally and economically limited for women to a greater extent than men. Even if a woman had access to a motorbike, it is reported that she would most likely need to find someone to accompany her. Of two relais located in the same commune (Gnibi), the man responded he uses a cooler and motorbike to cover 21 villages, while the woman stated that she mainly serves the households in town. Others noted that women have significant constraints on their time, and that maternity leave and marriage presented real barriers for female relais in completing their work. Relais are not provided supplies necessary to preserve a cold chain, so when their work requires it, they must access their own cooler and ice. Some trainings have supplied coolers to relais, but they still must purchase ice. For women, this requires economic independence or reliance on and support of their male partners. Gender norms also paint a picture of women as having less courage and physical strength than men–with some participants questioning whether women can wrangle and manage small ruminants. While some women and some men indicated that women can do whatever men can do, most agreed that, nonetheless, it is not culturally sanctioned for female relais to do much of what male relais do.

*Lower barriers to the ND VVC foster greater motivation among women than men*. Men and women’s engagement as relais also differ by value chain; most relais involved in PPR are men, whereas most relais for ND are women. This is, in part, based on cultural beliefs and norms, which limit women’s ability to fully engage in the management of small ruminant care. Furthermore, poultry is widely regarded as a woman’s space, except in the case of large commercial poultry operations where men are predominant. Thus, women also report significantly greater motivation than men to serve as relais in the ND VVC, due to the benefits they derive as poultry producers. Some actors indicate that women relais are more likely to stay in their position and are more reliable than men. Participants also indicate that despite a lack of monthly salary, the potential to earn small amounts of income and improve the health of their poultry motivates women to serve as poultry animal health workers. Conversely, men tend to minimize the work associated with such low-earning positions, emphasizing the lack of incentives. Importantly, even though participants indicate that women have *fewer* constraints in serving as ND relais and more motivation, constraints still exist: demands on women’s time for household responsibilities are significant in the early morning and late evening, which are also the times that are best/easiest to vaccinate poultry.

#### Lower nodes of the VVCs in Kaffrine: Livestock holders

During KIIs and especially FGDs, livestock keepers shared their different experiences and perceptions about men’s and women’s roles in Senegalese society, how vaccines are used, by whom, and what barriers exist across the region. The application of GITA analysis yielded the following themes.

*Men have greater power over vaccination use*, *but women want acknowledgment*. Most men keep cattle, horses/donkeys, and small ruminants (mostly sheep), while women keep poultry and small ruminants. Men and women did not agree on how responsibilities for large and small ruminants vary by gender. Men claim to have main responsibilities for ruminants for instance, while women claimed they are the ones who mostly take care of small and sometimes large ruminants, on a daily basis–even if they formally belong to the husbands. Women see themselves as suitable livestock keepers–in some communities claiming to know better than men how many animals a household has, what diseases affect which animals, and what the animals eat. Women’s ability to manage small and large-stock is limited, however, by their inability to travel at night to seek veterinary assistance, locate animals that did not come home at night, etc. Some men described women as unable to secure transport (a cart) to go to the vet, needing a man in order to do so; others disagreed. Women find it easy to keep poultry, which is compatible with their normal household work and, when things go well, can be quite lucrative. Men tended to agree with that and demonstrated little to no interest in poultry, for reasons already mentioned–small poultry is associated with women’s space–but also because mortality is perceived to be high and not worth their effort.

*Knowledge of vaccination is uneven*, *especially among uneducated women*. Knowledge of livestock diseases is mixed, with some respondents reporting feeling hopeless in the face of livestock diseases that they do not recognize or are unable to treat. While most men and women don’t know the exact names of diseases, symptoms are known and well-described. Barriers to animal health identified among men and women were similar; these included not only poor access to veterinary services, but additional barriers such as lack of feed, lack of pasture (grazing land) and water, and lack of enclosed spaces to keep animals safe. Confusion about vaccination exists, underscored by a common inability to distinguish between injections for treatment and those for prevention. Anecdotes about part of the herd dying after vaccination are often repeated, fostering confusion, especially in communities who have had no direct training. Some farmers prefer to seek services from a traditional animal health practitioner or healer, stating that traditional veterinary medicine is just as effective as western medicine. Women, in particular, shared a lack of understanding about how the system can serve them and, specifically, about how vaccines work. Women who know how to vaccinate chickens are those who have received training, a skill many focus group participants demanded.

*Confidence in the vaccine distribution system is lacking*, *especially among some groups*. The first line of access for the health care of small ruminants is almost always the technical livestock agent or a private veterinarian rather than a relais. In some areas the assigned veterinarian is well-known and respected, while in others, people lament their tardiness and delays, including in vaccine administration. If the veterinarian is not available when an animal is sick, men report that they may seek help from the community relais. In some cases, men and women have confidence in the relais, but in others, for small ruminants, and especially among men where the relais is a woman, they lack confidence that the relais can help them. Especially in underserved locations, it is not uncommon to consult traditional healers also for animals.

*Remoteness of communities/households is a key barrier and intersects with poverty*. Geographical distance from veterinary services was identified as a key factor preventing communities from accessing veterinary services, especially in the rainy season when roads are washed out. Among keepers of small ruminants, the distance to vaccination locations (often referred at ‘vaccination parks’) and the associated costs of transporting animals are a key constraint. Poverty among livestock keepers exacerbates geographical distance and represents an added barrier for many families. The lack of willingness and/or ability of relais to serve them exacerbates their disadvantage. Remote households without young family members who can travel with animals to vaccination parks or veterinary posts are among the most vulnerable.

*The vaccination system often fails transhumant households*. Vaccination campaigns occur prior to the start of annual migration of livestock, primarily cattle and mall ruminants, to grazing areas (transhumance), which typically occurs at the beginning of the rainy season. However, vaccination delays are common, as noted earlier, because of overall procurement and distribution issues. This has real consequences for mobile pastoral communities, whose animals are difficult to reach once herds start moving north. Attitudes among sedentary populations about transhumant livestock practices were mixed. In some settled communities (largely Wolof), Fulani herders were blamed for spreading diseases through shared water points. Community members indicated that herders did not vaccinate their livestock, and that when confronted to present vaccination cards, the Fulani failed to do so. In contrast, another settled community described Fulani herders as exemplars–receiving informal training on vaccination, having an exceptional relationship with the technical livestock agent, and regularly administering the vaccines.

## Discussion

While our findings reveal the important role of education and touch on possible ethnic differences in some areas, the two factors that most define the experience of actors along the VVCs are geographic isolation (especially when compounded with poverty) and gender. Firstly, remote households and communities–where remoteness can be defined as distance from the vaccination points and other services–have low access to vaccines regardless of the VVC (PPR or ND vaccines) and the type of vaccine. This disadvantage is deeper among households with fewer economic means and animals. Secondly, women are generally less likely to participate in the vaccine value chains when compared to men, as users of vaccines and as providers of livestock services and vaccines. A further important qualification becomes apparent. When it comes to men, their remoteness and poverty are likely to characterize their degree of involvement no matter the type of vaccine. In contrast, women appear to experience different degrees/types of barriers according to whether they are in the PPR or ND VVCs, the type of vaccine and associated administration modality.

We thus propose a characterization of gendered barriers within VVCs, by exploiting the advantages from our comparative approach. Not only do we analyze vaccines for two different livestock species (small ruminants and poultry) and diseases (ND and PPR), but also, within the vaccination system against ND, we compare the VVCs for two different vaccines (I2 and ITA-new), which are distributed according to the public and private system respectively.

Let us start by comparing livestock species. In Senegal, ruminants (large and small) are vaccinated, at least during the annual vaccination campaigns, in vaccination parks, thus requiring livestock keepers to physically move their animals to a location for vaccination. While women are the ones mostly taking care of small ruminants, there are cultural and social barriers to their being able to move these livestock to the vaccination parks, thus reducing their ability to access vaccines. Instead, women reported lower barriers and greater motivation to engage in the ND VVCs (both). This is partly because, in the case of poultry, the onus is upon vaccinators to go to the houses of livestock keepers and vaccinate there. Women also represent most poultry keepers and can make more autonomous decisions about those livestock.

A second difference is with respect to the type of vaccine–specifically the regulations governing the administration of different types of vaccines in Senegal. The higher formal educational requirements for the administration of live as opposed to inactivated vaccines mean that community vaccinators, such as relais or trained community members, are not allowed to administer PPR/H. Because of persistent gender inequalities in formal education and in access to jobs, in Kaffrine only 4 out of the 22 livestock technical agents–the vaccinator at the lowest node qualified to give the PPR/H vaccine–are women. This technical aspect of the vaccine reinforces the sharp gender differences across the studied VVCs, resulting in women dominating the vaccination of poultry, but being largely absent from vaccination of small ruminants.

The third difference is due more specifically to the distribution system. In the public distribution system, the vaccine is provided free by the state and the livestock keepers only pay a service fee to the providers. In the private distribution system, instead, livestock keepers bear the entire cost of the vaccine, unless the vaccine is partly funded and made available through development projects. To the extent that women are over-represented among poorer and more vulnerable smallholders, any failure in the public distribution system will exacerbate gender inequities in the distribution of vaccines. Even within the poultry sector, where women are most autonomous, many smallholders wait for the annual campaign that distributes the I2 vaccine and may face additional barriers in accessing vaccines if they must rely on the privately provided ITA-new vaccine.

Our meta-analysis thus shows that i) women face different degrees of barriers according to whether they are vaccine users, vaccinators at community level, or actors at higher node, and ii) the relevance of such barriers is a function of three dimensions within VVCs. This is visually conveyed in Table 2). When we look at women both as vaccine users and as vaccinators, we note similar gender differences along the livestock species and type of VVC: high barriers within the VVC for small ruminants’ vaccine and low barriers within poultry’ vaccine systems. A minor difference emerges when comparing the public and the private distribution system for the ND vaccine, since the cost barriers in the latter are a bit higher.

**Table 2:** Degree of barriers faced by women at different nodes of the VVC, by animal species and vaccine type.

	*Women as vaccine users*	*Women as vaccinators at community level*	*Women at higher nodes*
Small ruminants			
PPR/H (public–live vaccine/injection)	High barriers	High barriers	Very high barriers
Poultry			
I2 (public–attenuated vaccine/drops)	Low barriers	Low barriers	Very high barriers
ITA-new (private—inactivated vaccine)	Low to medium barriers	Low barriers	Very high barriers

At the higher node of the VVCs (last column), however, gender barriers remain high no matter what the type of VVC. With few exceptions (e.g. a woman at the Head of Regional Livestock Services for Kaffrine), there are far fewer women than men operating at higher level of command in the animal health system.

Our qualitative data suggest that such barriers are not just due to differential access to higher education–since the gender gap in tertiary enrolment in Senegal is reducing in this respect–but to self-selection into what are culturally perceived as "gendered” disciplines and career tracks, as well as social norms that inhibit women’s perceived success in”men’s” spaces. Social norms have until now reduced the percentage of girls choosing veterinary training–although our informants signal that this gender gap is narrowing. Beyond the choice of educational field, there exist important and persistent differences in social expectations about men’s and women’s appropriate roles in the family and in the workplace. Our interviews and focus group discussions with both men and women pointed to the existence of widely shared social norms, which limit women’s ability to make careers in fields that require physical mobility, strength and flexibility, and are, for these reasons, perceived to be more suitable to men. Women may thus have the qualifications, but not the family and social acceptance to move physically from one location to another to take up a better job position within the system. Changes in social norms would need to occur simultaneously at different levels, to influence expectations about what a girl can study, where a woman can live, when and where a woman can move independently, and what a wife and a mother can do as a profession. Future research may be needed on what types of change—in social norms or in institutions and structures—are most likely to have an impact on gendered barriers at different nodes in the VVCs.

## Conclusion

An efficient and equitable provision of livestock vaccines at the community level benefits the livelihoods of smallholder farmers, ultimately enhancing the food security and the well-being of their families. When vaccine distribution systems focus on diseases in poultry or small ruminants, women may benefit to a greater extent, given their greater economic dependence on these livestock species. In this paper we have employed a gender and intersectional analytical tool, the GITA, to map and dissect the gendered complexity of the VVCs of ND and PPR in Senegal; and to identify barriers that women and other groups may face within the vaccine distribution system. Results from this work indicate that the various experiences of individuals as vaccine users or providers are most defined by location and remoteness as well as gender; whereas they appear to be determined to a lesser degree by other social stratifications of age, ethnicity, and livelihood. In addition, the experience of women within these systems is defined to a much greater extent that that of men by the dynamics of the vaccine system itself. Differences according to the livestock species, regulation of vaccine administration, and vaccine distribution systems are more relevant for understanding barriers within livestock vaccination systems faced by women rather than by men. Our findings show the added value of utilizing qualitative data and nuanced thematic analysis to investigate gendered barriers within VVCs as well as a comparative approach that provides insights about gender differences not only within the nodes of a VVC, but also between VVCs for different livestock species and diseases.

It is important to note that the findings are based on our data collection methods, which rely on participants’ responses rather than on direct behavior observation. Other data collection methods may add to our current understandings of how socio-economic status, livelihood, or ethnicity interact and affect individual access to vaccines and the ability to benefit from the vaccine distribution system. However, it is worth noting that in the Kaffrine region people are predominantly rural and very poor, and most of them appear to face similar difficulties accessing vaccines, including geographical, institutional, and structural barriers. With such little variation, it is not surprising that socio-economic status does not emerge as a discerning characteristic of our population. Instead, barriers to vaccine knowledge, acceptance, and access are higher and more likely to be encountered by women livestock owners. Innovative, value chain specific solutions, which distinguish between the distinct needs of women according to location and animal production system, should be sought along all the nodes of the VVCs.
